# Mechanical and Durability Properties of Latex-Modified Hybrid Fiber-Reinforced Roller-Compacted Rapid-Set Cement Concrete for Pavement Repair

**DOI:** 10.3390/ma14143981

**Published:** 2021-07-16

**Authors:** Su-Jin Lee, Hyung-Jin Shin, Chan-Gi Park

**Affiliations:** 1 Department of Architectural Engineering, Keimyung University, Daegu 42601, Korea; sjlee@gw.kmu.ac.kr; 2 Rural Research Institute, Korea Rural Community Corporation, Ansan 15634, Korea; shjin@ekr.or.kr; 3 Department of Regional Construction Engineering, Kongju National University, Yesan 32439, Korea

**Keywords:** mechanical properties, durability, hybrid fiber, jute fiber, pavement repair, roller-compacted rapid-set cement concrete, structural synthetic fiber

## Abstract

This study evaluated the mechanical properties and durability performance of latex-modified hybrid fiber-reinforced roller-compacted rapid-set cement concrete (LMHFRCRSC) for emergency repair of concrete pavement. Experimental parameters included the blend ratio of the hybrid fiber, which comprised natural jute fiber (0–0.2 vol.%) and structural synthetic fiber (0–2 vol.%). The mechanical performance of LMHFRCRSC of various blend ratios was evaluated in terms of compressive, flexural, and splitting tensile strength. Durability assessment included chlorine ion penetration and abrasion resistance measurements. Compressive and flexural strength values of 21 and 3.5 MPa, respectively, were the set targets after 4 h of curing; a compressive strength of 35 MPa, a flexural strength of 4.5 MPa, a splitting tensile strength of 4.2 MPa, and chloride ion penetration of 2000 C or less were required after 28 days of curing. Our test results confirmed that all mix proportions satisfied the target values, regardless of the blend ratio of the hybrid fiber. Specifically, the mechanical performance of the concrete improved as the blend ratio of the structural synthetic fiber increased. With regard to durability, a greater amount of jute fiber, a hydrophilic fiber, enhanced the concrete’s durability. Additionally, incorporating jute fiber of 0.6 kg/m^3^ provided excellent chlorine ion penetration resistance. The optimal blend ratio for the hybrid fiber was natural jute fiber at 0.6 kg/m^3^ and structural synthetic fiber at 13.65 kg/m^3^ (mix: J0.6 + P13.65); with this mix proportion, a chloride ion penetration amount of 1000 C or less and maximum mechanical performance were achieved.

## 1. Introduction

Roller-compacted concrete (RCC), which exhibits little to no fluidity, does not have the consistency required for compaction, unlike general concrete, and thus is compacted by external vibrators such as a vibration roller and vibration press compactor [1,2,3]. Roller-compacted concrete pavement (RCCP), as a drier concrete, allows construction projects to proceed without interruption, as it sets rapidly without the need for reinforcement. Thus, this concrete is more economical compared to other concrete products [4,5,6]. RCCP has a low water-to-cement ratio compared to general concrete pavement; there is little moisture evaporation, which minimizes the likelihood of cracking due to shrinkage on evaporation. Therefore, RCCP offers the advantages of enhanced strength and durability over the long term [1,2,3,4,5,6].

If some of the constituent materials of the RCC are replaced with rapid-set cement and latex, it may be possible to further enhance the durability and workability simultaneously, thereby allowing the modified material to be used for emergency repair of concrete pavement [1,2]. However, most roller-compacted rapid-set cement concrete uses only 5% latex, which is less than the 10–15% commonly used in general emergency pavement repair materials [1,2]. Thus, despite being more economical, the performance and durability of rapid-set concrete for rapid repairs are somewhat compromised with respect to the resulting compressive strength, flexural strength, and permeability of the end product [1,2]. As such, a method is needed to overcome the reduction in mechanical performance and durability to exploit the material advantages of the RCC even if a small amount of latex is used. Crack control can potentially enhance both the mechanical properties and durability of concrete [7,8,9,10]. In particular, there is a possibility that cracks may occur in the concrete due to vibrations generated during compaction and mixing of the vibrating roller [11]. Therefore, it is necessary to control such cracks. However, when using fiber as a reinforcement material for crack control, the performance outcome will depend on the fiber type and its specifications [12,13,14,15,16]. For example, existing studies on concrete using single-fiber reinforcement have indicated that its performance is reduced compared to concrete incorporating 10–15% latex.

Another option would be to maximize the effect of fiber reinforcement using a hybrid fiber [16,17,18]. Hybrid fiber improves the performance of concrete by simultaneously controlling the occurrence and growth of micro- and macro-cracks [17,18,19]. This approach has been described in many studies [17,18,19].

This study used a hybrid fiber blend of structural synthetic fiber and natural jute fiber to minimize crack formation and spread in the concrete. Here, the structural synthetic fiber was designed to control for macro-cracks and the natural jute fiber was added to minimize micro-crack development. Structural synthetic fiber, as a concrete reinforcement material, is known to improve the compressive and tensile strength of concrete, as well as its corrosion and crack resistance [20,21,22]. For pavements, it is important to ensure that the reinforcement material is corrosion-resistant, particularly for pavement in which corrosion is likely due to freezing and thawing, as well as the use of de-icing materials in the winter. Jute has been shown to provide excellent crack control and an overall improvement in durability when added to concrete as a non-structural fiber. Recently, it has been widely used in Korea to control shrinkage cracks in concrete pavements, slabs, and parking lots [23]. Therefore, in this study, structural synthetic fibers and natural jute fibers were selected as materials for improving the performance of latex-modified hybrid fiber-reinforced roller-compacted rapid-set cement concrete (hereinafter referred to as LMHFRCRSC) for emergency repair of concrete pavements in consideration of the characteristics of concrete pavements exposed to corrosive environments such as de-icing materials.

Here, we evaluated the effects of varying the blend ratio of the hybrid fiber (consisting of structural synthetic fiber and natural jute fiber) on the performance of LMHFRCRSC for emergency repair of concrete pavement.

## 2. Materials and Methods

### 2.1. Materials

**Figure 1:**
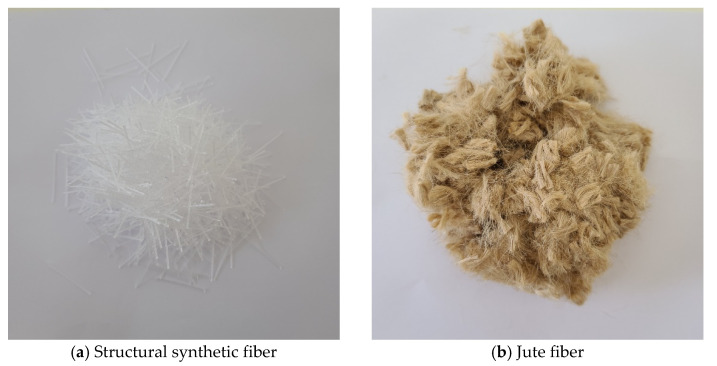
Studied fibers.

Table 1 shows the physical and chemical properties of the rapid-set cement (Jungang Polytech Co., Ltd., Yangsan, Gyeongnam, Korea) used in this study. As a coarse aggregate, crushed aggregate with a density of 2.618 g/mm^3^ and a maximum dimension of 13 mm was used. The fine aggregate consisted of river sand with a density of 2.58 g/mm^3^. Styrene butadiene latex (Jungang Polytech, Co., Ltd., Yangsan, Gyeongnam, Korea, hereinafter referred to as SB latex) was used in this study; its properties are shown in Table 2. The natural jute fiber and structural synthetic fiber (NYCON Materials Co., Ltd., Asan, Chungnam, Korea) were employed as reinforcement materials (Table 3 and Figure 1).

### 2.2. Mix Proportions

In the case of the concrete pavement repaired using the rapid-set cement, the American Association of State Highway and Transportation Officials, Road Transportation Bureau of each state in the United States [24] and the Korea Expressway Corporation [25] prescribe a minimum curing time of 4 h as the traffic opening time, in which the repair must meet a compressive strength target value of 21 MPa or more and a flexural strength of 3.5 MPa or more. After 28 days of curing, the required values are a compressive strength of 35 MPa, flexural strength of 4.5 MPa, and splitting tensile strength of 4.2 MPa. Given that the goal of this study was to develop an LMHFRCRSC blend for rapid concrete repairs, the set targets for our concrete mixture were as follows: a compressive strength of 21 MPa or more and flexural strength of 3.5 MPa or more after 4 h of curing; and a compressive strength of 35 MPa or more, flexural strength of 4.5 MPa or more, and splitting tensile strength of 4.2 MPa or more after 28 days. In terms of the durability, chlorine ion permeation ASTM C1202 tests [26] were conducted to measure the permeability of the concrete blends, as this parameter has the greatest negative effect on the service life of concrete pavement according to the standards of the Korea Expressway Corporation. Thus, a passing charge amount of 2000 C or less at 28 days was set as the target.

In this study, a mixture that satisfies the performance of the emergency repair concrete of the Korea Expressway Corporation was selected using previous research results [1,2]. The mixing ratio was modified to enable roller compaction using this mix proportions.

Therefore, a mix ratio that satisfies the target performance was set so that even a mix proportion that does not contain fibers can be applied to the field as an emergency repair material.

To secure a concrete blend capable of roller compaction that met the performance targets, the water-to-concrete (W/C) ratio of the blends was set to 0.28. Latex was added in a 5% solidity ratio to the cement weight. The reinforcement fibers were incorporated at 0–2 vol.% for the synthetic fiber and 0.1–0.2 vol.% for the natural jute fiber. Finally, the optimal blend ratio of the hybrid fiber was determined to optimize the mechanical performance and durability of the LMHFRCRSC, such that maximum strength was achieved along with a low chlorine ion permeation amount of 1000 C or less (considered to be very low). The blend ratios of the LMHFRCRSC examined in this study are summarized in Table 4.

### 2.3. Manufacturing of the Test Specimens

**Figure 2:**
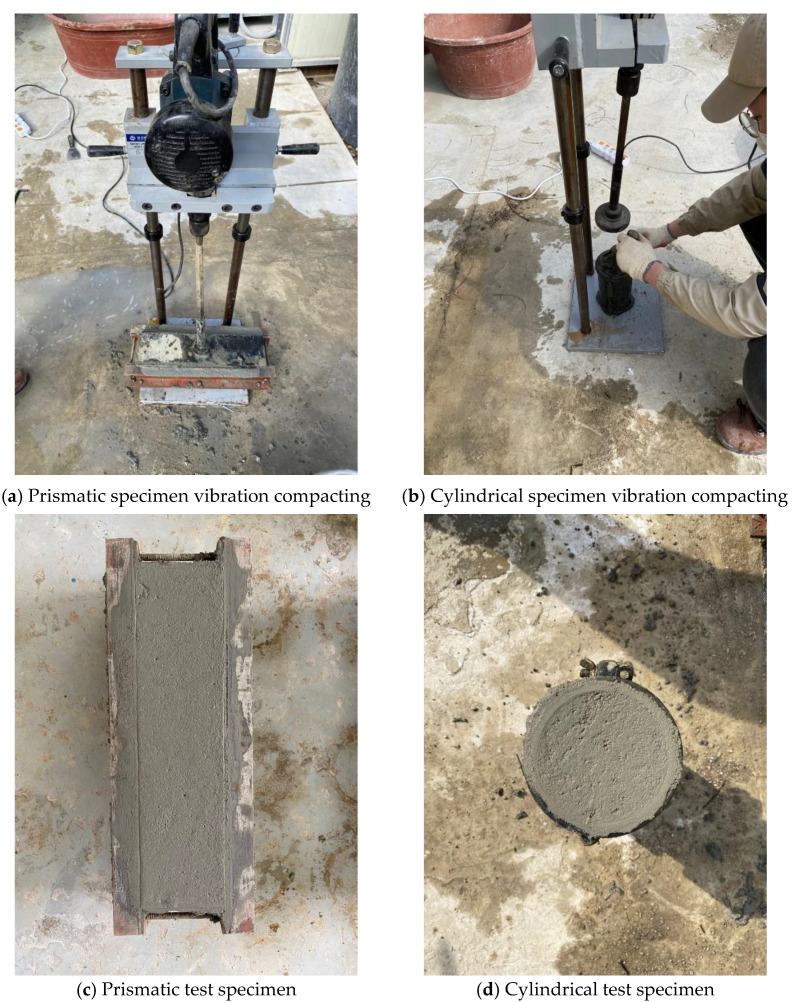
Manufacturing process of specimens using roller compacting vibrating machine.

The test specimens of the LMHFRCRSC for emergency repair of pavement were manufactured using a press compactor to simulate actual roller compaction. The specimens were based on a three-layer compaction configuration similar to general concrete in which the LMHFRCRSC was placed on the floor and subjected to vibration compaction for 30 s; the final layer underwent press vibration compaction to finish the surface. Figure 2 shows the specimen produced by the press vibration compactor and the press vibration compaction used in this study.

### 2.4. Test Methods

#### 2.4.1. Compressive Strength Tests

Compressive strength tests were performed in accordance with ASTM C39 standards [27]. A test specimen with a diameter of 100 mm and height of 200 mm was made and cured at 23 ± 2 °C and 58% relative humidity for 4 h and 24 h. The form was removed 4 h and 1 day later, and the specimen was then water-cured at 23 ± 2 °C for 28 days. Each test was performed after 4 h, 7 days, and 28 days of curing. Test specimens for evaluating compressive strength were manufactured twice in batches of three at each curing age, resulting in a total of six specimens for evaluating compressive strength at each curing age. In this study, the compressive strength was indicated as the average value of the six specimens for each curing age.

#### 2.4.2. Splitting Tensile Test

Splitting tensile tests were conducted in accordance with the ASTM C496 standard [28]. A test specimen with a diameter of 100 mm and height of 200 mm was made and cured at 23 ± 2 °C and 58% relative humidity for 4 h and 24 h. The form was removed 4 h and 1 day later, and the specimen was then water-cured at 23 ± 2 °C for 28 days. Each test was performed after 4 h, 7 days, and 28 days of curing. Test specimens for evaluating splitting tensile strength were manufactured twice in batches of three at each curing age, resulting in a total of six specimens for evaluating splitting tensile strength at each curing age. In this study, the splitting tensile strength was indicated as the average value of the six specimens for each curing age.

#### 2.4.3. Flexural Tests

Flexural tests were conducted in accordance with ASTM C496 [29]. A 100 × 100 × 400 mm³ test specimen of the prismatic type was made and cured at 23 ± 2 °C and 58% relative humidity for 4 h and 24 h. The form was then removed and the specimen was water-cured at 23 ± 2 °C. Each test was performed after 4 h, 7 days, and 28 days of curing. Test specimens for evaluating flexural strength were manufactured twice in batches of two at each curing age, resulting in a total of four specimens for evaluating flexural strength at each curing age. In this study, the flexural strength was indicated as the average value of the four specimens for each curing age.

#### 2.4.4. Chloride Ion Penetration Tests

The chloride ion penetration test was performed as follows. A test specimen with a diameter of 100 mm and height of 200 mm was made and cured at 23 ± 2 °C and 58% relative humidity for 4 h and 24 h; the form was then removed and the specimen was water-cured at 23 ± 2 °C for 28 days. After curing, the specimens were cut to a height of 50 mm. The cut specimens were placed in a vacuum desiccator and maintained in a vacuum for 3 h. The desiccator was then opened, the specimens were immersed in distilled water, and the vacuum was reapplied for another hour. The specimens were then removed from the desiccator. Each specimen was mounted onto the test cell filled with 3.0% NaCl solution on one side and 0.3% NaOH solution on the other side [26,30]. Testing was done according to the ASTM C1202-94 [26] “Standard Test Method for Electrical Indication of Concrete’s Ability to Resist Chloride Ion Penetration”. Each test was performed after 4 h and 28 days of curing. Test specimens for evaluating chloride ion penetration were manufactured twice in batches of two at each curing age, resulting in a total of four specimens for evaluating chloride ion penetration at each curing age. In this study, the chloride ion penetration was indicated as the average value of the four specimens for each curing age.

#### 2.4.5. Abrasion Tests

Abrasion tests were conducted in accordance with the ASTM C944 standard [31]. A test specimen with a diameter of 150 mm and height of 60 mm was made and cured at 23 ± 2 °C and 58% relative humidity for 24 h. The form was removed 24 h later, and the specimen was then water-cured at 23 ± 2 °C for 28 days. The test was performed after 28 days of curing. Test specimens for evaluating abrasion were manufactured twice in batches of two at each curing age, resulting in a total of four specimens for evaluating abrasion. The weight of each specimen was measured before and after the abrasion test, and the weight loss was determined as the amount of abrasion. The test result was expressed as the amount of loss per unit volume, that is, the amount of volume loss (g/cm^3^). In this study, the abrasion test results were presented as the average value of the four specimens.

## 3. Test Results

### 3.1. Compressive Strength

**Figure 3:**
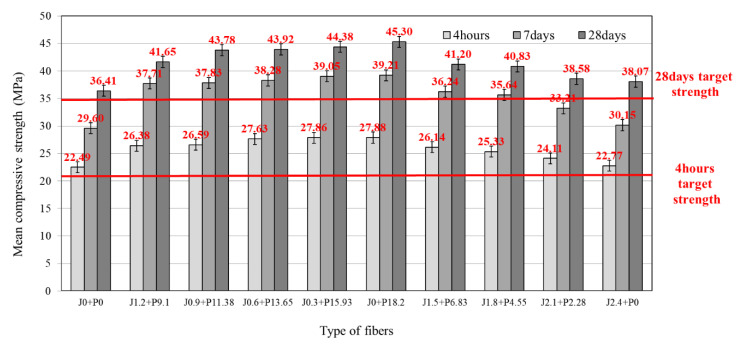
Compressive strength of LMHFRRCRC.

Figure 3 shows the results of the compressive strength tests with respect to the LMHFRCRSC mixing ratio. Under press vibration compaction, because the internal structure of the concrete is dense, any increase in compressive strength is readily apparent. The tests showed that all mixtures achieved the target compressive strengths of 21 MPa at 4 h and 35 MPa at 28 days, regardless of the blend ratio of the hybrid fiber. The (J0 + P0) blend, consisting of 0% jute (J) and 0% synthetic fiber (P), also satisfied the target performance in the basic experiments and was selected as the LMHFRCRSC control blend. Reviewing the results of incorporating the fiber, it was confirmed that the blend using the structural synthetic fiber alone at 18.2 kg/m^3^ or 2.0 vol.% (J0 + P18.2) significantly enhanced the compressive strength compared to (J0 + P0), whereas the blend using the jute fiber alone at 2.4 kg/m^3^ or 0.2 vol.% (J2.4 + P0) showed the same strength performance as the blend without any fiber reinforcement. Furthermore, in the case of the hybrid fiber-reinforced blend, the compressive strength improved as the incorporation rate of the structural synthetic fiber increased; however, increasing the amount of jute fiber in the hybrid fiber blend reduced the compressive strength of the concrete mixture. Thus, the higher the proportion of structural synthetic fiber in the blend, the greater the compressive strength.

### 3.2. Flexural Strength

**Figure 4:**
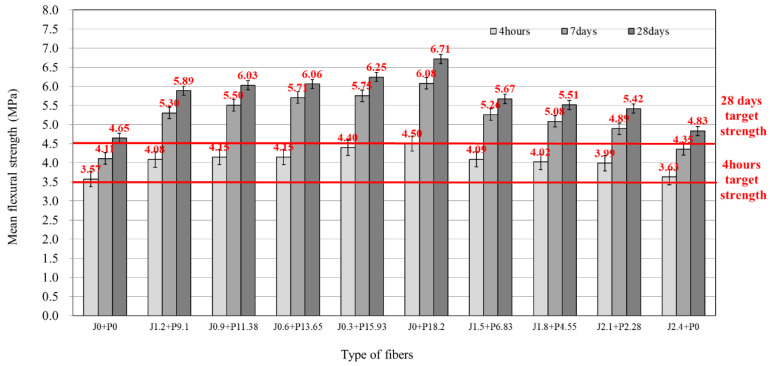
Flexural strength of LMHFRRCRC.

Figure 4 shows the results of testing the flexural strength according to the blend ratio of the hybrid fiber of the LMHFRCRSC. The target flexural strength of 3.5 MPa at 4 h of age was satisfied for all fiber mixing ratios. Similar to the compressive strength test results, the flexural strength increased with the proportion of structural synthetic fiber in the mixing ratio of the hybrid fiber. Likewise, the flexural strength decreased as the proportion of jute fiber increased. The flexural strength increase at 4 h of age was 1.0 to 25.9% with the fiber compared to the blend not incorporating fiber (i.e., the control blend, J0 + P0). Moreover, the blend using structural synthetic fiber alone (J0 + P18.2) at 18.2 kg/m^3^ (2.0 vol.%) showed the maximum flexural strength with an increase of 25.9% compared to the blend without fiber. The blend using the jute fiber alone (J2.4 + P0) showed the lowest increase, 1.0%, such that there was little to no effect on flexural strength with respect to the control. Furthermore, the flexural strength at 28 days showed similar tendencies: an increase in flexural strength with structural synthetic fiber in the blend and a reduction in flexural strength with jute fiber in the blend. However, the results indicated that all mixtures satisfied the target strength of 4.5 MPa at 28 days. The reason why the mechanical performance satisfied the target value, regardless of the mixing ratio of the hybrid fiber or whether fiber was incorporated, is that the structure of the concrete became dense due to press vibration compaction simulating vibration roller compaction. However, the increase in strength depended on the mixing ratio of the hybrid fiber, as the mechanisms by which the two types of fibers (synthetic vs. jute) reinforce the concrete are different. The structural synthetic fiber has sufficient specifications (e.g., shape ratio) such that the concrete may sufficiently absorb the fracture energy for better ductility. In contrast, the jute fiber is effective in controlling cracking as opposed to providing reinforcement.

### 3.3. Splitting Tensile Strength

**Figure 5:**
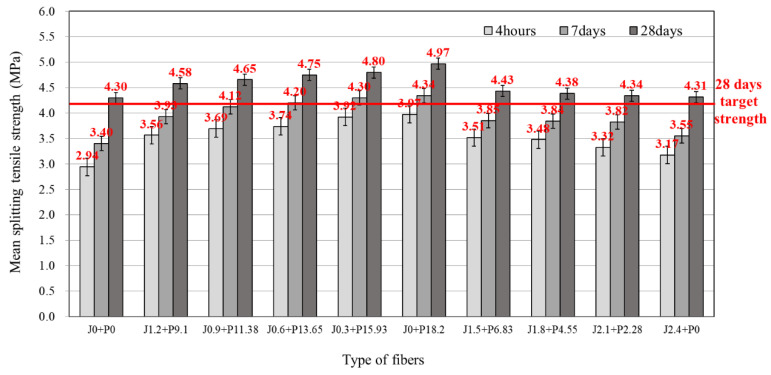
Splitting tensile strength of LMHFRRCRC.

Figure 5 shows the splitting tensile strength test results according to the mixing ratio of the LMHFRRCRC. As a result of the test, the target splitting tensile strength after 28 days of curing of 4.2 MPa was satisfied for all mixtures. The press vibration compaction conducted in this study was effective in increasing the strength by making the concrete structure more dense, and it improved the overall mechanical performance. As the proportion of structural synthetic fiber increased, so too did the splitting tensile strength. Among the blends, mixtures incorporating structural synthetic fiber in the amount of 13.65–18.2 kg/m^3^ satisfied the target strength after 7 and 28 days of curing. The structural synthetic fiber improved the flexural strength of the concrete as a reinforcement material. Therefore, blends using structural synthetic fiber alone (J0 + P18.2) provided the highest splitting tensile strength, whereas blends incorporating the maximum amount of jute fiber alone (J2.4 + P0) showed the lowest splitting tensile strength values. The improvement in splitting tensile strength using the hybrid fiber ranged from 0.3 to 15.6% compared to that of not incorporating either fiber (J0 + P0).

In summary, the jute fiber had no significant positive effect on the mechanical performance of the concrete. Therefore, to improve the mechanical performance of the LMHFRCRSC, the proportion of the structural synthetic fiber in the blend ratio of the hybrid fiber should be increased, with structural synthetic fiber alone providing the maximum effect.

### 3.4. Chloride Ion Penetration

**Figure 6:**
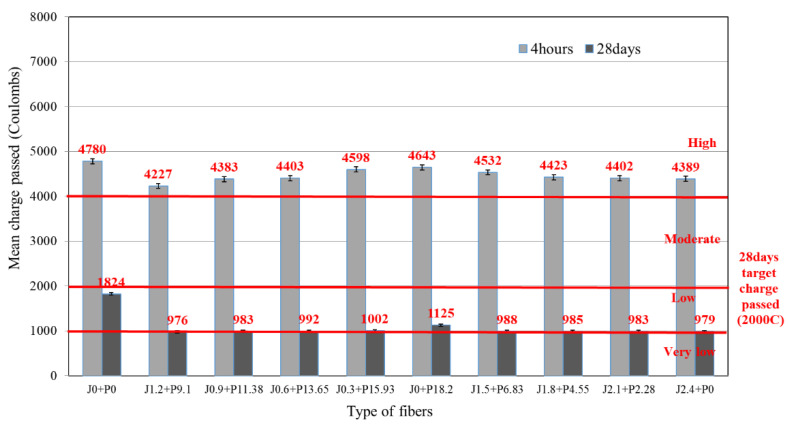
Chloride ion penetration of LMFRRCRC.

Figure 6 shows the results of the chlorine ion permeation resistance tests for the LMHFRRCRC. After 4 h of curing, all mixes had a passing charge amount of 4000 C or more, considered to be in the high range by ASTM standards. However, at 28 days of age, all mixes showed a target passing charge amount of 2000 C or less, which is considered to be in the low range for the permeability standard. The fibers, latex, and concrete effectively formed an impermeable layer inside the concrete and made the concrete structure more dense with press vibration compaction. Here, the reinforcing hybrid fiber was able to control the cracks inside the concrete, which actively reduced the permeability of the concrete. It is important to emphasize the effect of the blend ratio of the hybrid fiber on the permeability; specifically, as the proportion of the structural synthetic fiber increased, the passing charge amount increased, whereas as the proportion of jute fiber increased, the passing charge amount decreased. Optimal results were achieved with a blend incorporating structural synthetic fiber at 13.65 kg/m^3^ and jute fiber at 0.6 kg/m^3^ (J0.6 + P13.65), which showed a permeability of 1000 C or less (very low) and had a low permeability corresponding to the passing charge amount in the range of 1000–2000 C if the proportion of the structural synthetic fiber increased. On the other hand, as the proportion of the jute fiber in the hybrid fiber blend increased, the passing charge amount decreased. This result is due to the material properties of the two fibers. The structural synthetic fiber, as a fiber with a hydrophobic surface, effectively increases the physical bonding surface by crimping the surface shape to improve the bonding force with the concrete. Nevertheless, the perfect bonding properties observed at the concrete–fiber interface also served to increase the concrete’s permeability. However, the jute fiber with a hydrophilic surface has a microscopic size, as well as improved bonding with the concrete to control micro-crack formation and propagation inside the concrete. Thus, the jute fiber reduces the permeability of the concrete. From these results, the jute fiber is more effective in reducing the permeability of the concrete than the structural synthetic fiber.

Generally, the chloride ion penetration test is an indirect permeability test, not a test directly indicating the permeability of concrete. Therefore, durability, which is affected by the permeability of concrete, requires additional tests such as those regarding the sulfate attack effect, etc. [32].

### 3.5. Abrasion Resistance

**Figure 7:**
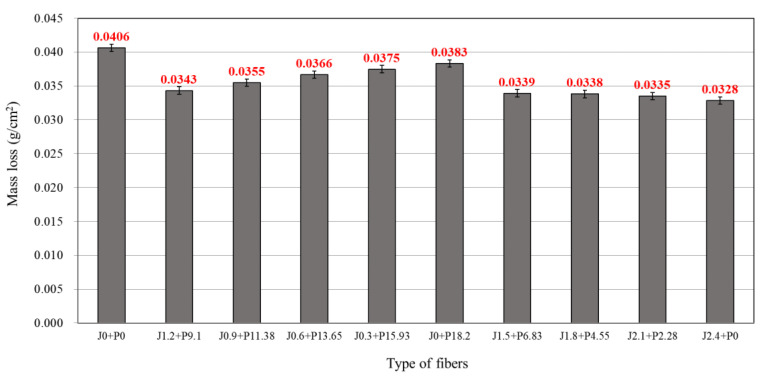
Abrasion resistance of LMHFRRCRC.

Figure 7 shows the mass reduction amount of the LMHFRCRSC measured by the abrasion test. The reduction in mass of the LMHFRCRSC produced by making the structure of the concrete dense via press vibration compaction was 0.04 g/cm^2^ for all mixtures, thereby confirming excellent abrasion resistance. Comparing the above results to the abrasion amount of the blend not incorporating fiber (the control, J0 + P0), it was shown that the abrasion resistance increased by about 5.7–19.3%. Regarding the effect of the blend ratio of the hybrid fiber, as the proportion of the jute or structural synthetic fiber increased in each blend ratio the abrasion amount increased and the abrasion resistance decreased. Particularly, the blend with a high proportion of structural synthetic fiber showed significant abrasion wear compared to that with a high proportion of jute fiber. This is because the jute fiber, as a micro-fiber, may be positioned close to the surface of the concrete where abrasion mainly occurs, providing direct resistance against abrasion. Reviewing the blend ratio of the hybrid fiber, which was decreased by 10% or more compared to the base blend of the control (J0 + P0), blending of the jute fiber at 0.6 kg/m^3^ or more provided the best results.

## 4. Conclusions

This study evaluated the effect of the blend ratio of a hybrid fiber comprising synthetic and natural jute fibers on the strength and durability of LMHFRCRSC mixtures for emergency repair of concrete pavement via roller compaction. The test results are summarized below.

The target values for the compressive, flexural, and splitting tensile strength after 4 h and 28 days of curing were satisfied for all mixtures considered. This result was simulated by actual roller compaction through press vibration compaction when producing the specimen to make the structure of the concrete dense. Press vibration enhanced the strength of the LMHFRCRSC. Furthermore, it was shown that as the proportion of the structural synthetic fiber in the blend ratio of the hybrid fiber increased, so too did the compressive strength; however, jute fiber reinforcement alone had no significant impact on the strength of the concrete. Thus, increasing the proportion of structural synthetic fiber improved the mechanical performance of the LMHFRCRSC.

The durability of the LMHFRCRSC was investigated with respect to the target values for chloride ion permeation and abrasion resistance; all mixtures met the target values, similar to the mechanical performance results. However, unlike the mechanical performance results, the durability improved as the proportion of jute fiber in the hybrid fiber blend increased. Specifically, at 28 days, a chlorine ion permeation resistance of 2000 C or less was achieved for all mixtures. Among these, the blend using 0.6 kg/m^3^ or more of jute fiber had a permeability of 1000 C or less (very low). This is because the jute fiber, as a hydrophilic fiber, adjusted to the occurrence and growth of micro-cracks through strong hydrogen bonding inside the concrete.

For abrasion resistance, the test results showed that as the proportion of jute fiber in the blend ratio of the hybrid fiber increased, the abrasion amount decreased. Jute fiber, as a hydrophilic micro-fiber, forms strong bonds with the cement matrix on the surface of the concrete, thereby suppressing the abrasion effect that otherwise occurs. Similar to the chlorine ion permeation resistance, the abrasion resistance improved by 10% or more when the blend ratio of the hybrid fiber incorporated 0.6 kg/m^3^ of jute fiber or more.

In summary, this study attempted to determine the optimal blend of hybrid fiber constituents for a LMHFRCRSC used for emergency repair of concrete pavement via roller compaction, with the goal of obtaining a material with excellent durability and meeting the mechanical performance targets required by ASTM standards. Specifically, a blend having very low permeability based on the passing charge amount and the highest strength was identified; this blend consisted of jute fiber at 0.6 kg/m^3^ and structural synthetic fiber at 13.65 kg/m^3^ (mix: J0.6 + P13.65).

## Figures and Tables

**Table 1:** Chemical composition of rapid-set cement.

SiO_2_ (%)	Al_2_O_3_ (%)	Fe_2_O_3_ (%)	CaO (%)	MgO (%)	K_2_O (%)	SO_3_ (%)
13 ± 3	17.5 ± 3	3>	50 ± 3	2.5>	0.21	14 ± 3

**Table 2:** Properties of styrene butadiene latex.

Solids Content (%)	Styrene Content (%)	Butadiene Content (%)	pH	Specific Gravity	Surface Tension (dyne/cm)	Particle Size (A)	Viscosity (cps)
30	34 ± 1.5	66 ± 1.5	11.0	1.02	30.57	1700	42

**Table 3:** Properties of fibers.

Property	Structural Synthetic Fiber	Natural Jute Fiber
Elastic modulus (GPa)	10	61
Tensile strength (MPa)	550	510
Density (g/mm^3^)	0.91	1.26
Fiber length (mm)	30	6
Fiber diameter (mm)	1	0.015

**Table 4:** Mix proportions LMHFRCRSC for pavement repair.

No. of Mix	W/C (%)	S/A (%)	Unit Weight (kg/m^3^)						
			W	C	S	G	Latex	Jute Fiber	Structural Synthetic Fiber
J0 + P0	28	55	112	400	1015	831	5	0.0	0.00
J1.2 + P9.1				396				1.2	9.10
J0.9 + P11.38				392				0.9	11.38
J0.6 + P13.65				388				0.6	13.65
J0.3 + P15.93				384				0.3	15.93
J0 + P18.2				400				0.0	18.20
J1.5 + P6.83				396				1.5	6.83
J1.8 + P4.55				392				1.8	4.55
J2.1 + P2.28				388				2.1	2.28
J2.4 + P0				384				2.4	0.00

## Data Availability

Not applicable.
